# Supplementary material for: A Novel Biplex Onchocerca volvulus Rapid Diagnostic Test Evaluated Among 3- to 9-Year-Old Children in Maridi, South Sudan
Source: Diagnostics (Basel). 2025 Feb 26;15(5):563. doi: 10.3390/diagnostics15050563 (PMC11898602; doi:10.3390/diagnostics15050563)
Supplement: Supplementary file 1 [file diagnostics-15-00563-s001.zip › diagnostics-3446309-supplementary.pdf]

Suplement Table S1: DDTD Biplex A rapid diagnostic test seroprevalence using different positivity assumptions per characteristic, incl. village, sex, ivermectin use, dermatitis, epilepsy and age group.

Factor	Level	DDTD Biplex A Prevalence T1 or T2 (% ;95%CI)	DDTD Biplex A Prevalence All T1 (% ;95%CI)	DDTD Biplex A Prevalence All T2 (% ;95%CI)	DDTD Biplex A Prevalence Only T1 (% ;95%CI)	DDTD Biplex A Prevalence Only T2 (% ;95%CI)
village	Gabat	2/50 (4; 0.7-14.86)	2/50 (4; 0.7-14.86)	0/50 (0; 0-8.89)	2/50 (4; 0.7-14.86)	0/50 (0; 0-8.89)
	KazanaI	30/50 (60; 45.2-73.27)	27/50 (54; 39.45-67.94)	14/50 (28; 16.67-42.71)	16/50 (32; 19.93-46.83)	3/50 (6; 1.56-17.54)
	KazanaII	28/38 (73.68; 56.61-86.02)	25/38 (65.79; 48.58-79.86)	17/38 (44.74; 28.99-61.52)	11/38 (28.95; 15.98-46.11)	3/38 (7.89; 2.06-22.48)
	Matara	18/51 (35.29; 22.8-50)	18/51 (35.29; 22.8-50)	9/51 (17.65; 8.87-31.36)	9/51 (17.65; 8.87-31.36)	0/51 (0; 0-8.73)
	Tarawa	13/50 (26; 15.08-40.61)	12/50 (24; 13.52-38.49)	4/50 (8; 2.59-20.11)	9/50 (18; 9.05-31.92)	1/50 (2; 0.1-12.01)
sex	Female	52/129 (40.31; 31.88-49.33)	48/129 (37.21; 28.99-46.2)	25/129 (19.38; 13.16-27.47)	27/129 (20.93; 14.47-29.16)	4/129 (3.1; 1-8.23)
	Male	39/110 (35.45; 26.73-45.21)	36/110 (32.73; 24.26-42.42)	19/110 (17.27; 10.98-25.92)	20/110 (18.18; 11.72-26.93)	3/110 (2.73; 0.71-8.35)
ivermectin	No	57/165 (34.55; 27.43-42.39)	53/165 (32.12; 25.19-39.9)	31/165 (18.79; 13.3-25.76)	26/165 (15.76; 10.73-22.42)	4/165 (2.42; 0.78-6.48)
	Yes	34/74 (45.95; 34.44-57.87)	31/74 (41.89; 30.71-53.92)	13/74 (17.57; 10.04-28.53)	21/74 (28.38; 18.8-40.23)	3/74 (4.05; 1.05-12.18)
dermititis	No	45/151 (29.8; 22.78-37.87)	43/151 (28.48; 21.58-36.49)	21/151 (13.91; 9.01-20.7)	24/151 (15.89; 10.64-22.93)	2/151 (1.32; 0.23-5.2)
	Yes	45/87 (51.72; 40.82-62.47)	40/87 (45.98; 35.36-56.96)	23/87 (26.44; 17.82-37.17)	22/87 (25.29; 16.85-35.94)	5/87 (5.75; 2.14-13.5)
epilepsy	No	89/235 (37.87; 31.71-44.44)	84/235 (35.74; 29.69-42.27)	42/235 (17.87; 13.32-23.51)	47/235 (20; 15.2-25.81)	5/235 (2.13; 0.79-5.17)
	Yes	2/4 (50; 15-85)	0/4 (0; 0-60.42)	2/4 (50; 15-85)	0/4 (0; 0-60.42)	2/4 (50; 15-85)
Age group	Age 3-6	56/147 (38.1; 30.32-46.5)	51/147 (34.69; 27.16-43.04)	23/147 (15.65; 10.38-22.76)	33/147 (22.45; 16.16-30.21)	5/147 (3.4; 1.26-8.17)
	Age 7-9	35/92 (38.04; 28.3-48.8)	33/92 (35.87; 26.33-46.61)	21/92 (22.83; 14.99-32.98)	14/92 (15.22; 8.87-24.56)	2/92 (2.17; 0.38-8.38)

T1 tests for the presence of Ov16 and OvOC3261 antibodies, and T2 for Ov33.3 and OvOC10469. Positivity assumptions include: “T1 or T2” one of the lines can be positive for the test to be positive; “All T1”, if T1 was positive the test was positive; “All T2”; if T2 was positive the test was positive; “Only T1”, a test was positive if only T1 and not T2 was positive; “Only T2”, a test was positive if only T2 and not T1 was positive.
